# Mining a Cathepsin Inhibitor Library for New Antiparasitic Drug Leads

**DOI:** 10.1371/journal.pntd.0001023

**Published:** 2011-05-03

**Authors:** Kenny K. H. Ang, Joseline Ratnam, Jiri Gut, Jennifer Legac, Elizabeth Hansell, Zachary B. Mackey, Katarzyna M. Skrzypczynska, Anjan Debnath, Juan C. Engel, Philip J. Rosenthal, James H. McKerrow, Michelle R. Arkin, Adam R. Renslo

**Affiliations:** 1 The Small Molecule Discovery Center, University of California San Francisco, San Francisco, California, United States of America; 2 Department of Medicine, San Francisco General Hospital, University of California San Francisco, San Francisco, California, United States of America; 3 The Sandler Center for Drug Discovery, University of California San Francisco, San Francisco, California, United States of America; 4 Department of Pathology, University of California San Francisco, San Francisco, California, United States of America; 5 Department of Pharmaceutical Chemistry, University of California San Francisco, San Francisco, California, United States of America; McGill University, Canada

## Abstract

The targeting of parasite cysteine proteases with small molecules is emerging as a possible approach to treat tropical parasitic diseases such as sleeping sickness, Chagas' disease, and malaria. The homology of parasite cysteine proteases to the human cathepsins suggests that inhibitors originally developed for the latter may be a source of promising lead compounds for the former. We describe here the screening of a unique ∼2,100-member cathepsin inhibitor library against five parasite cysteine proteases thought to be relevant in tropical parasitic diseases. Compounds active against parasite enzymes were subsequently screened against cultured *Plasmodium falciparum*, *Trypanosoma brucei brucei* and/or *Trypanosoma cruzi* parasites and evaluated for cytotoxicity to mammalian cells. The end products of this effort include the identification of sub-micromolar cell-active leads as well as the elucidation of structure-activity trends that can guide further optimization efforts.

## Author Summary

Diseases like malaria and sleeping sickness are caused by tropical parasites and represent a major cause of mortality and morbidity in the developing world. A pragmatic approach to discover new drugs for these diseases is to search for drug leads among existing small molecule collections generated in the for-profit pharmaceutical industry. In this study, we searched for new drug leads among a collection of small molecules donated by Celera Genomics. This collection of molecules was originally developed to inhibit a class of human enzymes (cathepsins) implicated in diseases like osteoporosis and psoriasis. Similar enzymes are also present in most tropical parasites, making this collection a logical place to search for new drug leads. The end result of this effort saw the identification of compounds that inhibit the growth of one or more tropical parasites and that will serve as good starting points for the development of new drugs for tropical parasitic diseases.

## Introduction

There is a critical need for new drugs to treat neglected tropical diseases [1], [2], [3], [4]. Current therapies are limited by inadequate efficacy, drug resistance, or toxicity. Chagas' disease, for example, remains the leading cause of heart disease in Latin America, with between 8 and 12 million people currently infected, and over 90 million at risk (as reported in WHO fact sheet No. 340, June 2010). Current therapy for Chagas' disease consists of the nitro heterocycles nifurtimox and benznidazole. Both drugs have frequent and serious side effects [5] limiting their efficacy, and each requires an extended (60–120 days) course of therapy. Furthermore, resistance to nifurtimox is emerging. Drug choices for those suffering from human African trypanosomiasis (HAT, sleeping sickness) are similarly poor, with organoarsenic derivatives (e.g. melarsoprol) still employed in the treatment of CNS disease despite a ∼5% rate of drug-associated mortality [6]. In the treatment of malaria, the artemisinin-based combination therapies (ACT) [7] are currently effective and well tolerated, but resistance to the partner drugs [8] and possibly to artemisinins as well [9] is emerging.

One pragmatic strategy to develop new antiparasitic drug leads is to focus on targets that are shared by multiple pathogens. Cysteine proteases play essential roles in numerous protozoan and helminth parasites, and are therefore appropriate targets for antiparasitic chemotherapy [10]. The Clan CA [11] cysteine protease cruzain (cruzipain) has been advanced as a potential drug target in *Trypanosoma cruzi*, the parasite responsible for Chagas' disease [12]. Cruzain is highly expressed in the intracellular amastigote stage of the parasite that is responsible for human disease. Cruzain deficient strains of the parasite fail to establish infection in immune-competent experimental hosts [13]. Because genetic deletion of the cruzain gene is not feasible, the target has instead been validated with small molecules. Hence, irreversible inhibitors of cruzain produce a characteristic swelling of the parasite Golgi compartment in *T. cruzi* parasites, leading to subsequent dysmorphic changes in both Golgi and endoplasmic reticulum, and ultimately rupture of parasite cells [14]. A number of cysteine protease inhibitors have been shown to arrest *T. cruzi* development within mammalian cells *in vitro*, and some have been shown to arrest or cure infection in mouse models of disease [15]. In *Trypanosoma brucei* parasites, the cysteine proteases rhodesain (brucipain) and a cathepsin-B like protease (TbCatB) have been advanced as potential drug targets [16], [17].

The erythrocytic malaria parasite produces a variety of proteases that perform the essential function of hemoglobin hydrolysis, whereby the parasite acquires its principal source of amino acids [18], [19]. We and others have shown that cysteine protease inhibitors block the hydrolysis of hemoglobin by erythrocytic parasites, causing the food vacuole to fill with undegraded hemoglobin, preventing parasite development, and indicating that cysteine proteases play an essential role in hemoglobin hydrolysis [20]. Among the various proteases that mediate hemoglobin hydrolysis are the cysteine proteases falcipain-2 and falcipain-3. Disruption of the falcipain-2 gene leads to a block in trophozoite hemoglobin hydrolysis [21]; the falcipain-3 gene could not be disrupted, strongly suggesting that this protease is essential for erythrocytic parasites [22]. The inhibition of cysteine protease mediated hydrolysis of hemoglobin is therefore a promising and active area of drug discovery research [23], [24], [25].

A concern in targeting parasite cysteine proteases with chemotherapeutic agents is the potential for toxicity due to the existence of homologues such as cathepsin B, K, S, and L in the mammalian host. This fear has to some extent been alleviated by pre-clinical experience with cysteine protease inhibitors targeting plasmodium and trypanosome parasites. Even compounds that are not significantly selective at the biochemical level have proven to be well tolerated in animals. Why might this be so? One possibility is that whereas mammalian cysteine proteases are a redundant gene family in which many discreet catalytic types occur in the same lysosome or vacuolar compartment, parasite enzymes – although sometimes part of a small gene family – tend to be less redundant [26]. A second factor is that the host-origin lysosomal cysteine proteases are present in mammalian organelles at millimolar concentrations, much higher than is thought to be the case for the parasite proteases [27], [28]. As such, it would be difficult to completely eliminate host cathepsin activity in the context of a short course of therapy targeting an acute infection. Nevertheless, a team of academic and industry researchers recently reported a series of nitrile-based cruzain inhibitors that display excellent in vitro selectivity for the parasite protease over mammalian cathepsins [29].

The cysteine protease inhibitor library screened in this study is unique and not commercially available. It is composed entirely of compounds synthesized by medicinal chemists at Celera Genomics in the course of various discovery campaigns targeting human cathepsins, primarily cathepsins K, S, and B [30], [31], [32], [33]. The library members are generally peptidic in nature and nearly all possess an electrophilic “warhead” that engages the active-site cysteine thiol reversibly or irreversibly through the formation of a C-S bond. The topic of irreversible enzyme inhibition continues to generate healthy debate, with reversible (even if covalent-reversible) modes of inhibition generally viewed as being preferable [34]. This position seems prudent in the case of chronic conditions where long-term safety in very large patient populations is of paramount importance (e.g., in osteoporosis). In the case of an acute infection however, irreversible inhibition is arguably advantageous, a view that has been advanced in recent review articles [35], [36]. In any event, the focus of the Celera group on chronic disorders like osteoporosis is reflected in the constitution of the inhibitor library, which is heavily biased towards reversible-covalent type inhibitors, most notably aminoacetonitriles and heterocyclic ketones. The library contains a much smaller number of irreversible inhibitors, such as fluoromethylketones [37] and vinylsulfones [30]. The objective of the study described herein was to mine this library of ∼2100 unique cathepsin inhibitors to identify target- and parasite-active lead compounds. This discovery paradigm seeks to leverage chemical entities already in existence for a new purpose, much akin to the re-purposing of approved drugs. An important advantage of re-purposing at the lead stage, however, is that it allows for subsequent optimization of drug properties specific to the antiparasitic indication.

## Methods

### Cysteine protease assays

Recombinant cruzain, rhodesain, TbCatB, falcipain-2 and falcipain-3 were expressed as described previously [38], [39], [40], [41], [42]. Inhibitor stock solutions were prepared in DMSO and screened at 0.1 µM for cruzain and rhodesain, 1 µM for TbCatB and 50 nM for falcipain-3 (0.5% DMSO in assay). For IC_50_ determinations, compounds were serially diluted in DMSO in the range of 25 µM – 0.001 µM for cruzain, rhodesain and TbCatB; and 5 µM – 0.06 nM for falcipain-2 and falcipain-3. Protease inhibition assays for cruzain and rhodesain were carried out in 96 well plate format as described previously [43]. Cruzain (4 nM), rhodesain (4 nM) or TbCatB (258 nM) was incubated with test compound in 100 mM sodium acetate, pH 5.5, containing 5 mM DTT and 0.01% Triton X-100 (buffer A), for 5 min at room temperature (the higher concentration of TbCatB was required due to its lower intrinsic activity against the fluorogenic substrate). Then buffer A containing Z-Phe-Arg-AMC (Bachem) was added to enzyme-compound mixture to give 10 µM substrate in a final assay volume of 200 µL. The rate of free AMC release was measured at excitation and emission wavelengths of 355 and 460 nm, respectively, with a microtiter plate spectrofluorimeter (SpectraMax M5, Molecular Devices) for 3 min. Percentage inhibition of test compound was calculated relative to the DMSO control (0% inhibition control). IC_50_ values were determined with Prism 4 software (GraphPad, San Diego, CA) using sigmoidal dose-response variable slope model.

Protease inhibition assays for falcipain-2 and falcipain-3 were carried out in 96 well plate format as described previously [42], [43] with modification for end-point readout. Falcipain-2 (21 nM) or falcipain-3 (78 nM) was incubated with test compound for 10 min at room temperature in 200 mM sodium acetate, pH 6, 10 mM DTT and 3.6% glycerol and 0.01% Triton X-100 (buffer B). Then buffer B containing Z-Leu-Arg-AMC (Bachem) was added to the enzyme-compound mixture to give 25 µM substrate in a final assay volume of 200 µL. After 15 min, 50 µL of 5 M acetic acid was added to each well to stop the reaction. End-point fluorescence was read in a spectrofluorimeter as above. The 0% inhibition control wells contained DMSO while 100% inhibition control wells contained 50 µM E-64 (Sigma). Percentage inhibition of test compound was calculated relative to the controls and IC_50_ curve fitting was performed with Prism 4 software as above.

### Trypanothione reductase assay

The enzyme inhibition assay was carried out in 96 well plate format as described previously [44] with modification in the sequence of substrate addition. Trypanothione reductase (3 mU/mL) was incubated with 10 µM test compound (1% DMSO in assay) for 30 min at room temperature in the presence of 50 µM DTNB (5,5′-Dithiobis(2-nitrobenzoic acid), 6 µM trypanothione, 40 mM Hepes pH 7.4 and 1 mM EDTA. Then 150 µM NADPH was added to give a final assay volume of 100 µL. The rate of TNB (2-nitro-5-mercaptobenzoic acid; yellow color) formation was measured in absorbance at 412 nm with a microtiter plate spectrofluorimeter (Flexstation, Molecular Devices). Percentage inhibition was determined with respect to the 0% inhibition control (1% DMSO).

### *T. brucei brucei* assay

The growth inhibition assay for *T. brucei brucei* was conducted as described previously [45]. Bloodstream forms of the monomorphic *T. brucei brucei* clone 427-221a were grown in complete HMI-9 medium containing 10% FBS, 10% Serum Plus medium (Sigma Inc. St. Louis Mo. USA), 50 U/mL penicillin and 50 µg/mL streptomycin (Invitrogen) at 37°C under a humidified atmosphere and 5% CO_2_. Inhibitor stocks were prepared in 100% DMSO and screened at 5 µM for percent inhibition values or serially diluted from 25 µM to 0.04 µM in 10% DMSO for IC_50_ determinations. 5 µL of each dilution was added to 95 µL of diluted parasites (1×10^4^ cells per well) in sterile Greiner 96-well flat white opaque culture plates such that the final DMSO concentration was 0.5%. The 0% inhibition control wells contained 0.5% DMSO while 100% inhibition control wells contained 50 µM thimerosal (Sigma). After compound addition, plates were incubated for 40 hours at 37°C. At the end of the incubation period, 50 µL of CellTiter-Glo™ reagent (Promega Inc., Madison, WI, USA) was added to each well and plates were placed on an orbital shaker at room temperature for 2 min to induce lysis. After an additional 10 min incubation without shaking to stabilize the signal, the ATP-bioluminescence of each well was determined using an Analyst HT plate reader (Molecular Devices, Sunnyvale, CA, USA). Raw values were converted to log_10_ and percentage inhibition calculated relative to the controls. IC_50_ curve fittings were performed with Prism 4 software as above.

### Jurkat cell cytotoxicity assay

Jurkat cells (clone E6-1) were grown in complete RPMI-1640 medium containing 10% FBS, 50 U/mL penicillin and 50 µg/mL streptomycin (Invitrogen) at 37°C under a humidified atmosphere and 5% CO_2_. For cytotoxicity testing, cells were diluted to 1×10^5^ per ml in complete RPMI-1640 medium. Test compound stocks were prepared in 100% DMSO and screened at 10 µM against cells (1×10^4^ cells per well) in sterile Greiner 96-well flat white opaque culture plates (0.5% final DMSO concentration). The 0% inhibition control wells contained 0.5% DMSO while 100% inhibition control wells contained 40 µM staurosporine. After compound addition, plates were incubated for 40 hr at 37°C. At the end of the incubation period, 50 µL of CellTiter-Glo™ reagent (Promega Inc., Madison, WI, USA) was added to each well and plates were placed on an orbital shaker at room temperature for 2 min to induce lysis. After a further 10 min incubation without shaking to stabilize the signal, the ATP-bioluminescence of each well was determined using an Analyst HT plate reader (Molecular Devices, Sunnyvale, CA, USA). Calculations of percentage inhibition and IC_50_ were performed similar to those in the *T. brucei brucei* assay.

### *P. falciparum* assay

The growth inhibition assay for *P. falciparum* was conducted as described previously [43]. W2-strain *P. falciparum* parasites (1% parasitemia, 2% hematocrit) were cultured at 37°C in human red blood cells under the atmosphere of 3% O_2_, 5% CO_2_ balance N_2_ in 0.2 mL of per well in medium RPMI-1640 supplemented with 10% human serum in duplicate 96-well flat bottom culture plates in the presence of inhibitors. Tested compounds were serially diluted 1∶3 in the range 10,000 – 4.6 nM, with maximum DMSO concentration of 0.1%. Following 48 hours of incubation, the medium was removed and the cells were fixed in 2% formaldehyde in PBS. Parasite growth was evaluated by flow cytometry on a FACsort (Becton Dickinson) equipped with AMS-1 loader (Cytek Development) after staining with 1 nM DNA dye YOYO-1(Molecular Probes) in 100 mM NH_4_Cl, 0.8% NaCl. Parasitemias were determined from dot plots (forward scatter vs. fluorescence) using CELLQUEST software (Becton Dickinson). IC_50_ values for growth inhibition were determined from plots of percentage control parasitemia over inhibitor concentration using GraphPad Prism software.

### *T. cruzi* amastigote assay

The growth inhibition assay for the intracellular *T. cruzi* was conducted in 96-well plate format as described previously [28]. Briefly, bovine embryo skeletal muscle (BESM) cells (2×10^3^/well), seeded overnight, were infected with CA-I/72 *T. cruzi* trypomastigotes (2×10^3^/well). Sixteen hours post-infection, test compounds (serial dilution concentrations starting from 20 µM) were added and assay plates were incubated for an additional 72 hours at 37°C with 5% CO_2_. Cells were then washed once in PBS, fixed with 4% paraformaldehyde and stained with DNA fluorescent dye DAPI (4′ 6-diamidino-2-phenylindole). The assay plates were then imaged in the IN Cell Analyzer 2000 (GE Healthcare) with the excitation and emission filters set of 350 nm and 460 nm, respectively, to detect DAPI signals. The feature extraction module in IN Cell Developer Toolbox 1.7 software was used to count host nuclei and parasite kinetoplasts by size difference.

### Cathepsin inhibitor library

**Figure 1:**
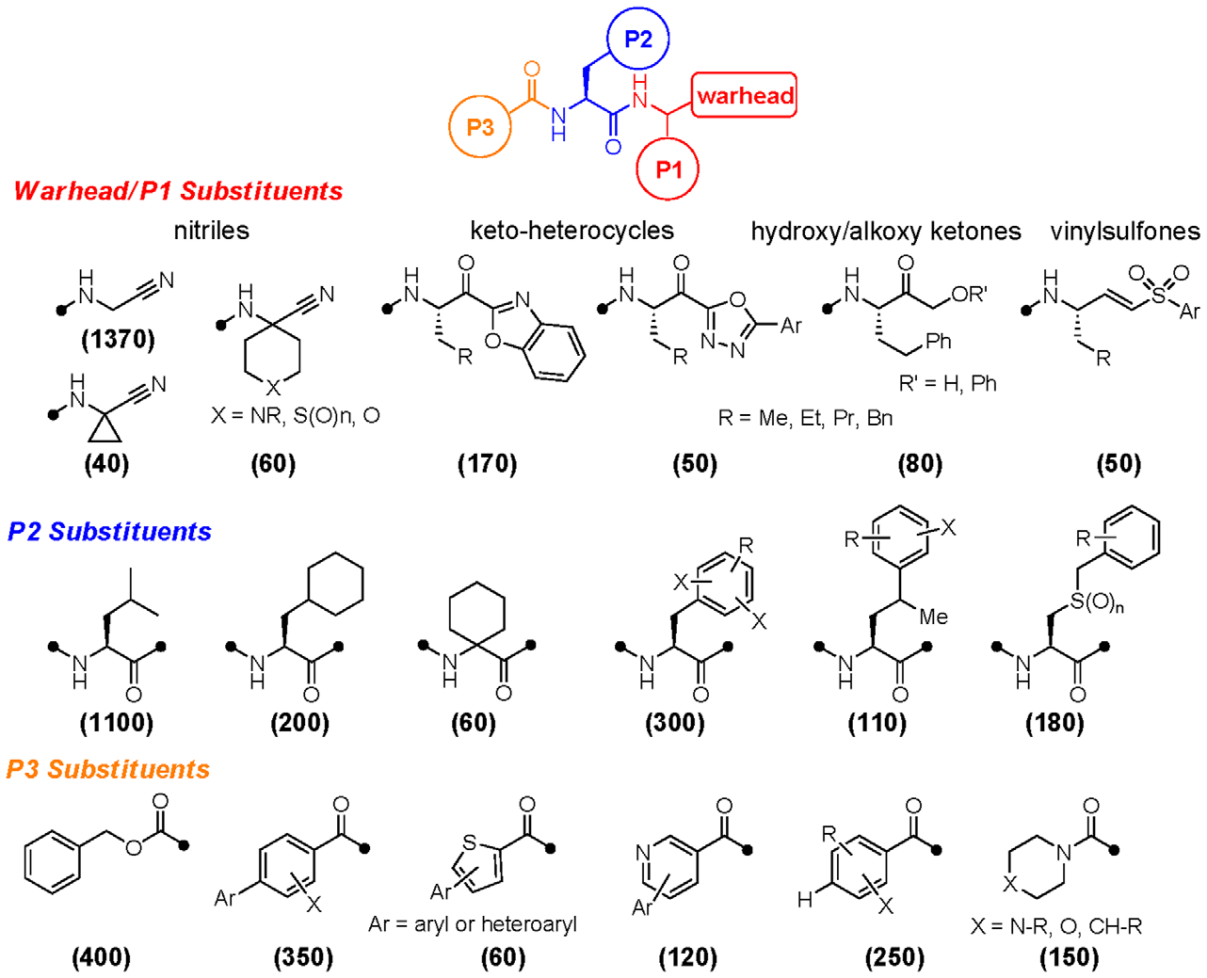
Structural composition of the cathepsin inhibitor library. Illustrated are significantly represented chemotypes at three positions: warhead/P1, P2, and P3. Indicated in parentheses are the approximate number of library members possessing each substructural chemotype.

The library was donated by Celera Genomics to the Sandler Center for Basic Research in Parasitic Disease at UCSF. The structural constitution of the library is summarized below (Figure 1). The vast majority of the compounds contain a biaryl or biheteroaryl ring system at the P3 position, a natural or unnatural amino acid at P2, and a thiol-reactive warhead function with or without a P1 substituent. Nearly three quarters of the library members are of the aminoacetonitrile warhead type and lack any P1 side chain; around 100 nitrile analogs possess a geminal (cyclic) substituent at P1. Other abundant inhibitor chemotypes include ∼170 ketobenzoxazoles, ∼50 ketooxadiazoles, and ∼80 α-hydroxy/alkoxy ketones. Around 50 irreversible inhibitors of the vinylsulfone type are present, as are a smaller number of other warheads. The majority of ketone and vinylsulfone warheads also contain a P1 side chain, and the majority of these are unbranched aliphatics of between two and four carbons, or are phenethyl.

The varieties of P2 side chains present in the library reflect the substrate specificity of the proteases being targeted and are heavily biased towards leucine and phenylalanine type side chains. A large number of analogs contain more extended P2 side chains (e.g., homo-phenylalanine or benzyl-cysteine), branching substituents and/or halogenations on the aromatic ring. About 60 analogs contain a geminal disubstituted (cyclic) moiety at P2. The majority of inhibitors are capped on their *N*-termini with benzoyl, acyl, carbamoyl (e.g., Cbz), or urea functions. Both aryl and heteroaryl P3 groups are represented, and many are highly substituted or bear an additional pendant aryl or heteroaryl ring substituent. Around 500 analogs contain such a biaryl or heterobiaryl P3 moiety and para-substituted biaryls are greater in number than meta- or ortho-substituted biaryls.

## Results

Evaluation of the cathepsin inhibitor library was envisioned to begin with a single-concentration screen which would be followed by analysis of actives in full dose-response and against cultured parasites. The determination of inhibition kinetics was deemed impractical given the size of the library. Still, we recognized the perils of comparing IC_50_ values between reversible and irreversible inhibitors, since inhibition by the latter is expected to be time dependent. We therefore first evaluated time-dependence of inhibition for a small but representative subset of the library (Table 1). Test compounds were pre-incubated with falcipain-3 for 0, 10, or 60 minutes prior to addition of a fluorogenic protease substrate. As expected, inhibitor types thought to act irreversibly (vinylsulfones, fluoromethylketones) showed time-dependent inhibition while those thought to confer reversible inhibition (e.g., nitriles) showed no time dependence. Even so, the irreversible inhibitors showed at most a ∼10-fold shift in IC_50_ value at the longer incubation times. We selected a 5 minute pre-incubation time for profiling the entire library, reasoning that comparisons of reversible and irreversible inhibitor potency in vitro is most meaningful with short incubation times, during the pre-covalent stage of inhibition by irreversible inhibitors.

**Table 1:** Effects of pre-incubation time on falcipain-3 IC_50_ values for inhibitors of different chemotype.

	Falcipain-3 IC_50_ (nM) pre-incubation time			
Compound	Chemotype	0 minute	10 minute	60 minute
1	Acyloxyketone	19	8	2
2	Aldehyde	5	4	4
3	Fluoromethylketone	97	30	8
4	Hydroxyketone	9	11	14
5	Ketoamide	20	19	22
6	Ketoheterocycle	8	7	6
7	Ketoheterocycle	8	9	10
8	Ketoheterocycle	10	8	7
9	Ketoheterocycle	3	3	3
10	Nitrile	41	44	50
11	Nitrile	5	3	3
12	Nitrile	15	16	17
13	Vinylsulfone	70	29	9

Single-point protease inhibition data were determined for the entire 2157 member library against the proteases cruzain, rhodesain, TbCatB, and falcipain-3. Falcipain-2 was not screened against but used only to test falcipain-3 hits. Protease inhibition was determined using standard functional assays [42], [43] employing fluorogenic substrates (Z-Phe-Arg-AMC or Z-Leu-Arg-AMC) and inhibitor concentrations of 50 nM (falcipain-3), 100 nM (cruzain and rhodesain), or 1 µM (TbCatB). To control for possible non-specific electrophilic inactivation of enzyme by test compounds, we also screened the library against trypanothione reductase, a non-protease that nonetheless contains catalytic active-site cysteine functionality. This latter assay was carried out according to an established method [44] but at a relatively high inhibitor concentration (10 µM) due to the expectation that the protease-targeted compound library would not potently inhibit trypanothione reductase.

**Figure 2:**
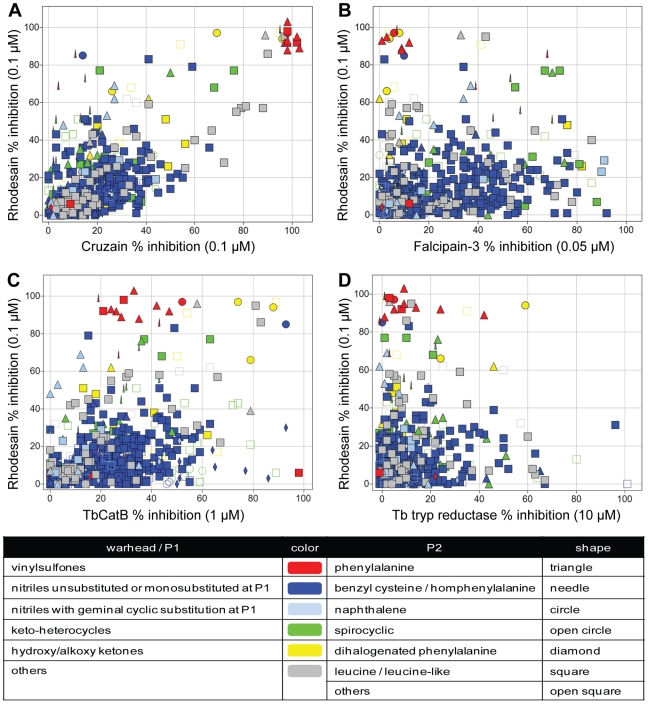
Comparing percent inhibition of compounds between cysteine proteases. Single-point inhibition data for rhodesain (Y-axis, % inhibition at 0.1 µM test compound) as compared to other parasite enzymes (X-axes), including: (A) cruzain (% inhibition at 0.1 µM), (B) falcipain-3 (% inhibition at 0.05 µM), (C) TbCatB (% inhibition at 1 µM), and (D) trypanathione reductase (% inhibition at 10 µM). Individual data points are colored according to warhead/P1 type as follows: vinylsulfones (red), nitriles unsubstituted or monosubstituted at P1 (blue), nitriles with geminal cyclic substitution at P1 (light blue), keto-heterocycles (green), hydroxy/alkoxy ketones (yellow) and others (grey). The shape of each data point corresponds to the P2 chemotype as follows: phenylalanine (triangle), benzyl cysteine or homphenylalanine (needle); naphthalene (circle), spirocyclic (open circle), dihalogenated phenylalanine (diamond), leucine/leucine-like (square) and others (open square).

Single-point screening data are presented as scatterplots and color/shape-coded according to warhead/P2 chemotype, as SAR at these positions provide the most useful information about substrate/inhibitor specificity (Figure 2). Hence, the cathepsin L-like proteases rhodesain and cruzain show a similar substrate-mimetic specificity, with the data points falling roughly along the diagonal of the scatterplot (Figure 2, panel A). The most potent inhibitors of these enzymes were vinylsulfones possessing Phe or similar (e.g. napthylalanine) P2 substituents. Also notable are a small number of rhodesain-selective analogs, many of which are characterized by the presence of more extended P2 side chains. The inhibition profile of falcipain-3 and TbCatB are distinct from that of either rhodesain or cruzain (panels B and C). Most of the potent inhibitors of falcipain-3 possess smaller Leu-like P2 moieties rather than the larger P2 side chains preferred by rhodesain (panel B). A small number of primarily ketone-based inhibitors were identified that significantly inhibit both proteases (panel B, green needles and green squares). Only a handful of library compounds inhibited TbCatB to a significant extent but strikingly, many of those that did possess large P2 moieties such as halogenated Phe/Tyr or napthylalanine (panel C, circles and needles). Furthermore, whereas the P2 halogenated Phe/Tyr analogs were selective for TbCatB over rhodesain, the P2 napthylalanine analogs inhibited both proteases potently. Conversely, P2 phenylalanine analogs bearing a vinylsulfone warhead proved highly selective for rhodesain over TbCatB (panel C, red triangles). Few inhibitors of trypanothione reductase were identified (even at 10 µM) and, as expected, no correlation between protease inhibition and inhibition of trypanothione reductase could be discerned (panel D). Thus, the single-concentration screening data provided useful insight into the substrate/inhibitor preferences of the various parasite proteases and set the stage for more careful profiling of specific compounds from the library.

**Figure 3:**
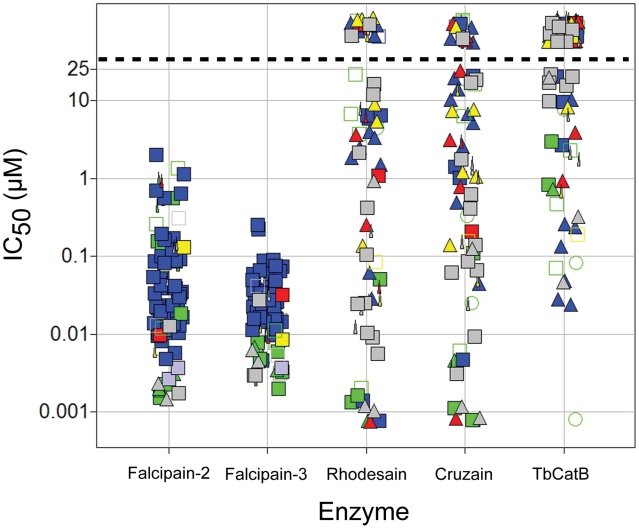
Fifty percent inhibition concentrations. IC_50_, (µM) against five parasite cysteine proteases as determined for library compounds that qualified as hits from the single point screens. Data points above the dashed line had no measurable IC_50_ (i.e., IC_50_>25 µM). Data points are colored and shaped by chemotype, as described in Figure 2.

**Figure 4:**
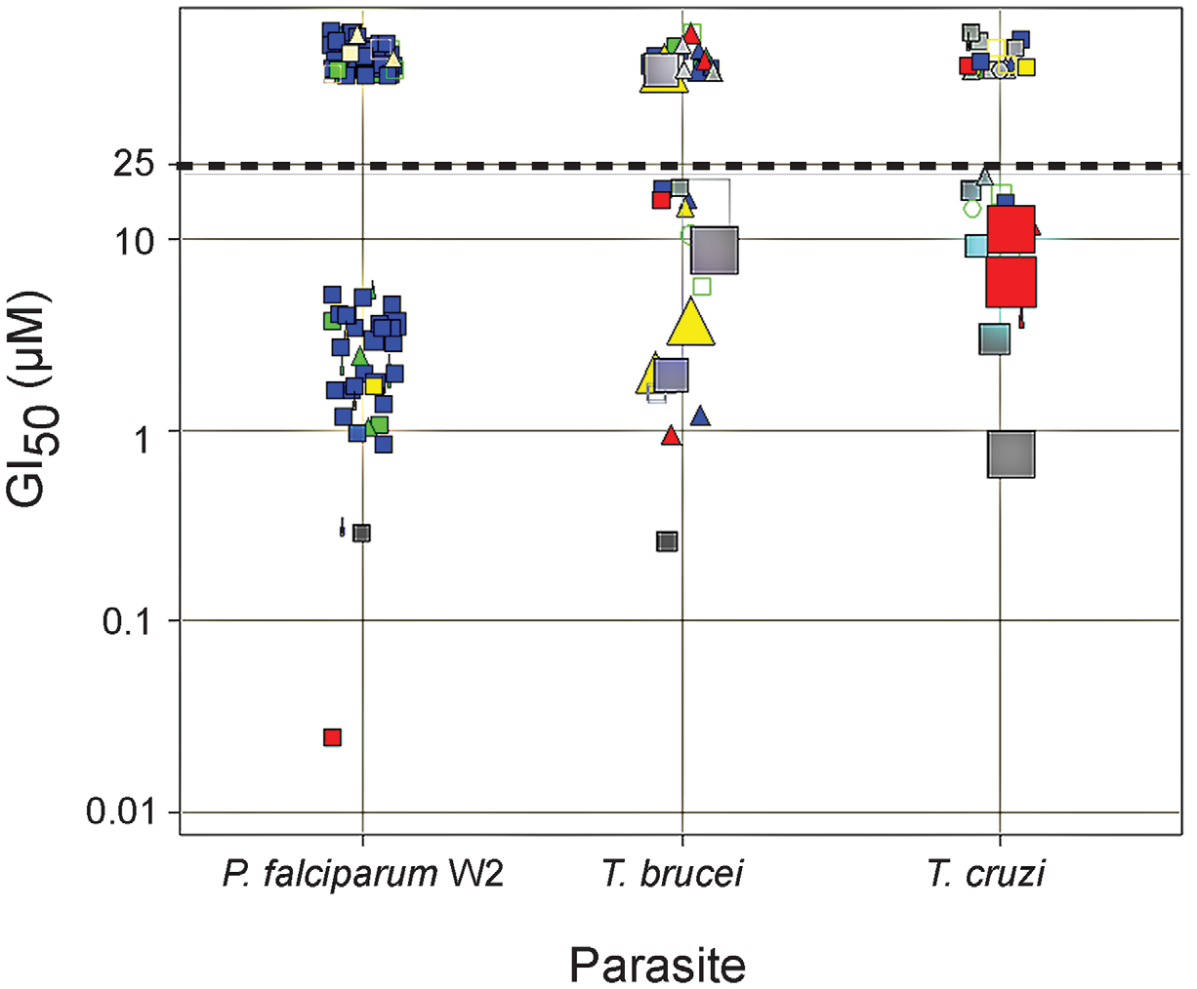
Growth inhibition of parasites. GI_50_ (µM) data for selected library members against cultured *P. falciparum* (W2 strain), *T. brucei brucei*, or *T. cruzi* parasites. Data points above the dotted line had no measurable GI_50_ (i.e., GI_50_>25 µM). Data points are colored and shaped by chemotype as described in Figure 2. The relative area of data points reflects cytotoxicity to mammalian cell lines (larger squares demoting increasing % growth inhibition relative to a 100% inhibition control). Cytotoxicity evaluations were performed in Jurkat cells for the *T. brucei* and *P. falciparum* actives (at 10 µM test compound) and in BESM cells for the *T. cruzi* actives (at 20 test µM compound).

The most potent inhibitors identified in the single-point screen (3–6% of the total library depending on enzyme) were subsequently evaluated in full dose-response to generate IC_50_ values for rhodesain, cruzain, TbCatB and falcipain-3. All falcipain-3 inhibitors were also tested against falcipain-2. The results are presented graphically in Figure 3, where it can be seen that a significant number of potent falcipain-2/3 and rhodesain/cruzain inhibitors, but many fewer potent inhibitors of TbCatB, were identified. Falcipain hits narrowly clustered at sub-micromolar IC_50_ with nitrile P1 warhead and leu-like P2 chemotypes predominating, while rhodesain/cruzain hits were more distributed in activity and chemical class. Accordingly, while more than one hundred falcipain-2/3 inhibitors were tested against cultured W2 *P. falciparum* parasites, only around thirty inhibitors of the trypanosome proteases were judged suitable for testing against *T. brucei brucei* and/or *T. cruzi* parasites. The entire library was examined for cytotoxicity to a mammalian cell line (Jurkat) at a concentration of 10 µM, and those compounds studied in the *T. cruzi* assay were also evaluated for toxicity (at 20 µM) toward the host BESM cells used in that assay. Parasite growth inhibition (GI_50_) and Jurkat/BESM cell cytotoxicity data are presented graphically in Figure 4. Nearly half of the falcipain inhibitors examined were found to inhibit parasite growth at low or sub-micromolar concentrations and without apparent cytotoxicity to Jurkat cells. A much smaller number of inhibitors were effective against *T. brucei brucei* or *T. cruzi* parasites and many of these were also cytotoxic to either Jurkat or BESM cells. The most promising parasite-active lead compounds are discussed in more detail below.

## Discussion

**Figure 5:**
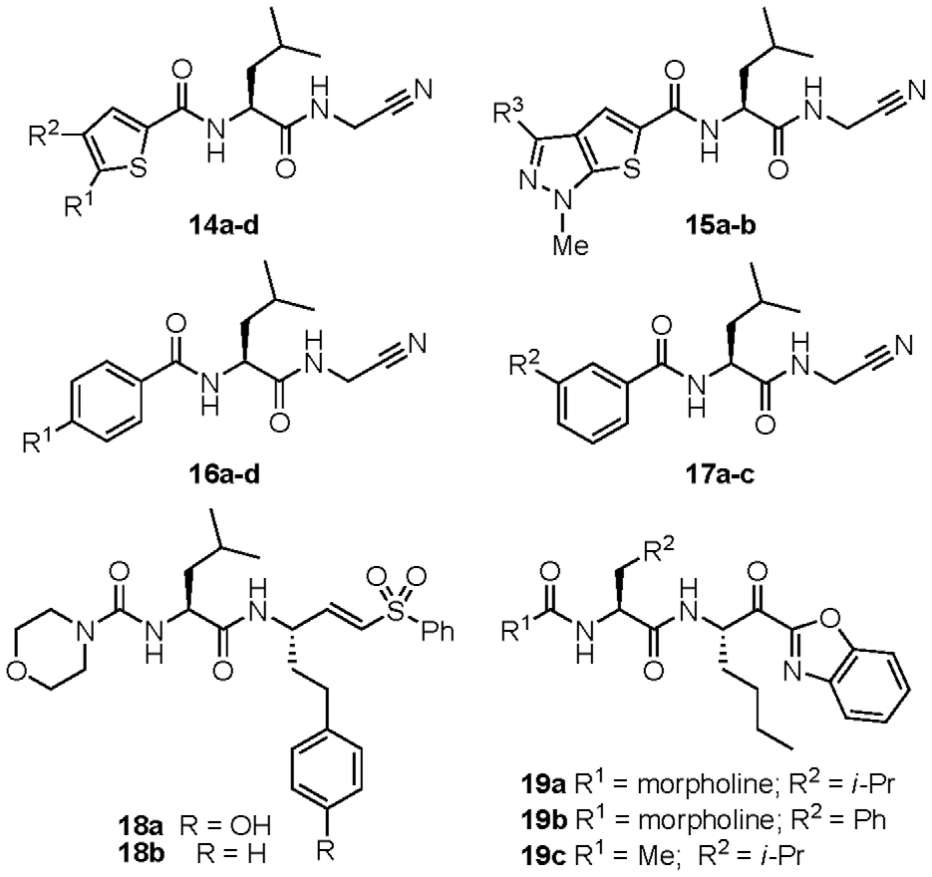
Anti-plasmodium lead compounds. Chemical structures of protease inhibitors discussed in the text. Associated enzyme inhibition and growth inhibition data is provided in tables 2–4.

**Figure 6:**
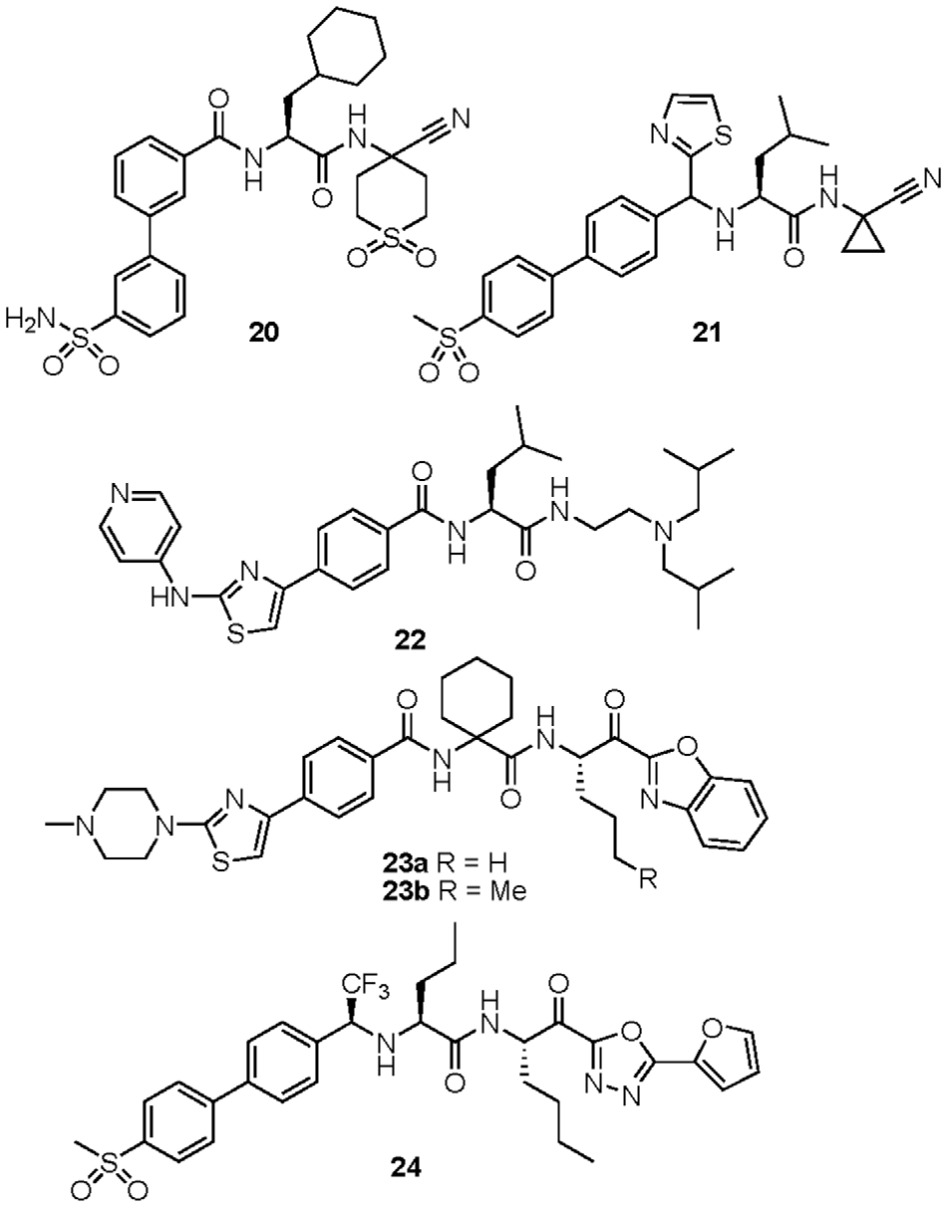
Anti-trypanosomal lead compounds. Chemical structures of protease inhibitors discussed in the text. Associated enzyme inhibition and growth inhibition data is provided in tables 4–5.

The parasite-active and non-cytotoxic analogs identified in the study can be considered as good candidates for future lead optimization efforts targeting parasitic indications. As starting points for such optimization, these compounds are likely more ‘advanced’ than compounds identified from typical screening libraries, as they were synthesized in the course of lead optimization campaigns targeting cathepsins. More than 85% of the library compounds have one or zero violations of Lipinski's ‘rule-of-five’ [46] and can therefore be regarded as having reasonable prospects for oral bioavailability. The structures and associated data are provided for ∼25 such lead compounds (Figures 5–6; Tables 2–5). The data presented in the tables includes historical inhibition values (Ki) against human cathepsin L and cathepsin B (data provided by Celera Genomics). As noted above, a significant number of compounds were identified that possess low- or sub-micromolar activity against cultured *P. falciparum* parasites. That so many plasmodium-active compounds were identified can be attributed to the known substrate preference of falcipains for leucine-like P2 side chains [47] which are very highly represented in the library (Figure 1). In fact, a leucine or leucine-like P2 side chain is found in nearly all of the anti-malarial lead compounds identified (compounds **14–21**, Figures 5 & 6).

**Table 2:** Selected anti-plasmodium lead compounds with associated biochemical and cell-based assay data.

				IC_50_ (µM)		Ki (µM)		GI_50_ (µM)	Cytotoxicity (% inhibition at 10 µM)
cmpd	R^1^	R^2^	R^3^	falcipain-2	falcipain-3	catL	catB	*P. falc.* W2	Jurkat cells
14a	furyl	furyl	—	0.020	0.029	0.076	16	1.5	<5%
14b	*o*-ClPh	H	—	0.0087	0.023	0.71	1.3	2.0	<5%
14c	H	*p*-MeOPh	—	0.018	0.025	0.35	4.2	2.7	<5%
14d	Cl	furyl	—	0.017	0.014	0.28	18	3.9	<5%
15a	—	—	CF_3_	0.050	0.015	0.15	54	3.5	<5%
15b	—	—	Ph	0.024	0.0052	0.11	90	>20	<5%

**Table 3 pntd-0001023-t003:** Selected anti-plasmodium lead compounds with associated enzyme and cell-based data.

		IC_50_ (µM)		Ki (µM)		GI_50_ (µM)	Cytotoxicity (% inhibition at 10 µM)
cmpd	R^1^/R^2^	falcipain-2	falcipain-3	catL	catB	*P. falc.* W2	Jurkat cells
16a	4-(MeSO_2_)Ph-	0.028	0.018	0.31	2.0	2.2	<5%
16b	3-(MeO)Ph-	0.075	0.067	0.68	1.6	3.5	<5%
16c	4-(HO)Ph-	0.078	0.028	4.3	5.2	4.0	<5%
16d	2-F-Ph-	0.068	0.085	48	20	>20	<5%
17a	2-(MeSO_2_)Ph-	0.017	0.016	2.2	18	0.84	<5%
17b	3-(MeS)Ph-	0.0088	0.028	0.11	5.7	3.0	<5%
17c	2-(Me)Ph-	0.026	0.067	0.22	24	>20	<5%

**Table 4 pntd-0001023-t004:** Selected anti-plasmodium lead compounds with associated enzyme and cell-based data.

	IC_50_ (µM)		Ki (µM)		GI_50_ (µM)	Cytotoxicity (% inhibition at 10 µM)
cmpd	falcipain-2	falcipain-3	catL	catB	*P. falc.* W2	Jurkat cells
18a	0.0076	0.040	0.003	0.44	0.02	<5%
18b	0.0036	0.029	0.0009	0.21	0.04	<5%
19a	0.0021	0.0083	0.026	0.125	1.2	<5%
19b	0.0044	0.0049	0.18	0.042	2.9	<5%
19c	0.066	0.017	0.61	0.92	4.8	<5%
20	0.0075	0.011	—	—	0.17	<5%
21	0.0020	0.0024	0.032	6.7	0.87	<5%

**Table 5:** Selected anti-trypanosomal lead compounds with associated enzyme and cell-based data.

	IC_50_ (µM)			Ki (µM)		GI_50_ (µM)		Cytotoxicity (% inhibition at 10 µM)
cmpd	rhodesain	TbCatB	cruzain	catL	catB	*T. brucei*	*T. cruzi*	Jurkat cells
19a	0.001	1.0	0.001	0.026	0.125	>25	20.0	<5%
19b	0.001	0.6	0.004	0.18	0.042	>25	10.9	<5%
22	0.64	>100	1.6	0.071	4.5	0.20	0.80	<5%
23a	3.9	0.07	0.03	1.1	0.32	4.2	19	<5%
23b	5.0	0.001	0.38	0.28	0.61	10.5	7.7	<5%
24	0.006	0.05	0.01	0.018	0.0016	7.0	7.6	<5%

Among the antimalarial lead compounds identified, a notable subset was found to possess a thiophene ring at the N-terminal (P3) position (Table 2). These analogs (**14a–d**) are substituted on the thiophene ring with one (**14b–d**) or two (**14a**) aryl or heteroaryl rings. Enzyme potencies for these analogs were in the mid-low nanomolar range while GI_50_ values were low single-digit micromolar. The structurally related thiophene analogs **15a–b** demonstrated potencies similar to **14a**, but while compound **15a** with a trifluoromethyl substituent was similarly effective against cultured *P. falciparum* parasites, the analogous phenyl substituted congener **15b** surprisingly was not. Inhibitors **14a–d** and **15a–b** were not cytotoxic to Jurkat cells at 10 µM, and inhibited human cathepsin L and B about 10 and 1000-fold less potently than the parasite proteases.

Analogs bearing para-biphenyl (**16a–d**) or meta-biphenyl (**17a–c**) P3 substituents represent a second class of anti-plasmodium lead compounds identified in this study (Table 3). The terminal aryl ring in these analogs appears to be insensitive to substitution pattern or type, with electron withdrawing or donating substituents well tolerated in various positions. Interestingly, the most cell-active analogs in this subset were **16a** and **17a**, both of which possess a methylsulfone substituent on the distal aryl ring. Indeed, analog **17a** is perhaps the most interesting lead compound in this set, with a sub-micromolar GI_50_ value, selectivity over the human cathepsins, and lead-like properties (MW∼425, clogP∼1.25) that would leave sufficient room to maneuver in a lead optimization campaign.

Two of the most potent analogs identified were vinylsulfone-based inhibitors of the type represented by **18a–b** (Table 4). In fact, compound **18a** was previously reported to be an inhibitor of *P. falciparum* growth, and structure-activity studies of similar compounds have been published [48]. A series of ketobenzoxazole-based analogs (**19a–c**) were also identified as potent inhibitors of falcipain-2/3 and *P. falciparum* growth in culture. Aside from the P1/warhead moiety, ketobenzoxazole **19a** is otherwise identical to the vinylsulfones **18a–b**, and indeed these compounds exhibit similar IC_50_ values against falcipain-2/3. Despite these similarities, the irreversible inhibitors **18a** and **18b** inhibited parasite growth at ∼100-fold lower concentrations than did the reversible inhibitor **19a**. It is not clear whether this effect is due to the nature of inhibition or other factors such as cell permeability, warhead inactivation (e.g. ketone reduction), or inhibition of other as-yet unidentified targets in the parasite.

Nitrile-based inhibitors **20** and **21** are also of significant interest as lead compounds as they represent the only reversible inhibitors identified that exhibited sub-micromolar GI_50_ values against *P. falciparum* parasites. Both inhibitors possess an *aliphatic* nitrile warhead geminally di-substituted at the P1 position and are thus structurally distinct from the *aromatic* nitriles (cyanopyrimidines) reported recently as potent inhibitors of falcipains [25]. The introduction of cyclic P1 moieties (as in **20** and **21**) has been reported previously [49] as a strategy to counter peptidase-mediated drug degradation *in vivo* and it seems plausible that these analogs may have been originally synthesized with such an objective in mind. Both analogs **20** and **21** have leucine or leucine-like P2 side chains and extended biphenyl moieties at P3, compound **21** possessing an interesting heterocyclic surrogate for the P2/P3 amide bond. The combination of reversible inhibition, low cytotoxicity, and nanomolar potency against falcipains 2/3 and whole parasites, recommend compound **20** in particular for further lead optimization studies.

Whereas a number of promising antimalarial lead compounds were identified in this study, very few compounds of clear merit were uncovered as leads for trypanosomal disease. In fact, the most potent antitypanosomal compound identified (**22**) appears not to be a protease inhibitor at all – having no apparent warhead function. While compound **22** did not inhibit the growth of Jurkat cells at 10 µM, it did significantly inhibit the growth of BESM cells (∼90% inhibition at 20 µM). It therefore seems likely that compound **22** confers its antitrypanosomal effect via a mechanism other than protease inhibition. While this is not in itself problematic, a mechanism of drug-like action (as opposed to non-specific toxicity) would need to be established before further pursuing compounds like **22**.

With respect to likely cysteine protease targets in *T. brucei*, RNAi knockdown of TbCatB but not rhodesain was shown to confer a phenotype similar to treatment with irreversible cysteine protease inhibitors [17]. Recently published data for small molecule inhibitors of TbCatB [50] also suggest that TbCatB is the more important drug target in *T. brucei*. Of significant interest then are ketobenzoxazole analogs like **23a** and **23b** which are potent (low-mid nM) inhibitors of TbCatB but only weak inhibitors of rhodesain (Table 5, Figure 6). Both compounds indeed inhibit the growth of *T. brucei brucei* parasites, although only at concentrations at least 100-fold greater than their *in vitro* IC_50_ values against TbCatB (low µM GI_50_ values vs. mid-low nM IC_50_ values). Ketooxadiazole analog **24** possesses excellent *in vitro* potency against both TbCatB and rhodesain, and like **23a/b** is effective against cultured *T. brucei brucei* parasites, but also at relatively high concentrations (GI_50_ = 7.0 µM). Ketobenzoxazole analogs **19a** and **19b** have quite the opposite selectivity profile of **23a/b** and are ∼1000 fold more potent inhibitors of rhodesain than TbCatB. That these compounds were not effective against *T. brucei brucei* parasites even at 25 µM is consistent with the hypothesis that TbCatB is the more important target in *T. brucei*. Thus, ketone-based inhibitors such as **23a–b** and **24** represent promising leads with regard to *in vitro* potency and selectivity (for **23a–b**) but would require further optimization for enhanced activity in cells. Possibly poor permeability and/or instability of the ketone warhead function in these compounds can explain their relatively modest activity against whole parasites.

**Figure 7:**
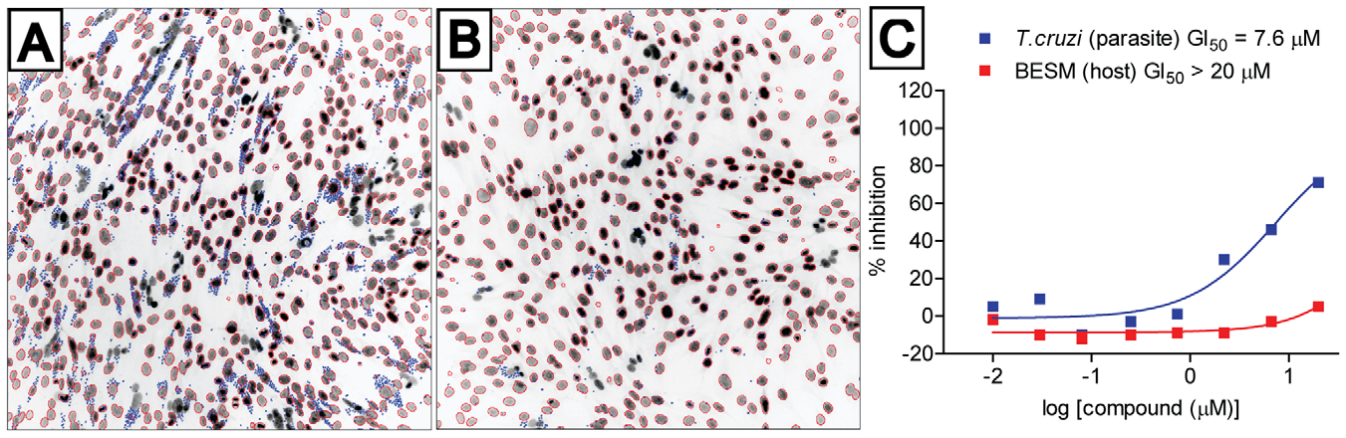
*T. cruzi* growth inhibition of compounds 24. Representative fluorescent images acquired with the IN Cell Analyzer 2000, taken with 20× objective magnification at Ex 350 nm/Em 460 nm, showing the host BESM nuclei (red outline) and surrounding smaller *T. cruzi* kinetoplastids (blue outline). (A) 1% DMSO as 0% inhibition control; (B) compound **24** at the highest assay concentration of 20 µM; (C) GI_50_ dose response plots of compound **24** against *T. cruzi* and cytotoxicity to host BESM cells.

A selection of potent cruzain inhibitors with diverse warhead types were selected for study in *T. cruzi* infected BESM cells using a microscopy-based high-content assay (Figure 7). Interestingly, the ketobenzoxazole analogs described above as active in *T. brucei brucei* were also among the most effective compounds against *T. cruzi* parasites. This is perhaps not surprising, since nearly all of these analogs are low nanomolar inhibitors of cruzain *in vitro* (Table 5). The most effective inhibitors were **23b** and **24**, but as was the case in *T brucei*, these compounds exert their effects on parasites only at much higher concentrations than are required to inhibit the target enzyme *in vitro*. As noted above, further chemical optimization of these leads for improved permeability and/or stability will likely be required to realize their full potential to affect parasite growth *in vitro* and *in vivo*. Also in favor of these compounds is their reversible-covalent nature of inhibition, which might be preferable to irreversible inhibitors with respect to the potential for in vivo toxicity and/or immunogenicity.

Comparisons of *in vitro* potency in biochemical assays with parasite growth inhibition is always problematic since factors such as cell permeability, compound stability, and sub-cellular localization are not always well understood and can vary with structure in unpredictable ways. Neither is it possible to rule out a role for as yet unidentified protease or non-protease targets as contributing to the observed parasite growth inhibition, since it is very likely that none of the protease inhibitors discussed herein is perfectly selective for the intended target(s). We have generally limited direct comparisons of potency to congeneric chemical series that inhibit by similar mechanisms (i.e., have similar warheads). For example, our discussion of protease targets in *T. brucei* is focused on reversible ketone-based inhibitors because the *in vitro* selectivity of such compounds is more likely to translate to the intracellular context than would selectivity data for irreversible inhibitors that exhibit time-dependent protease inhibition. We have similarly chosen not to make overreaching conclusions about the relative potency of compounds against parasite and human proteases, as the latter data were generated at an earlier time in a different laboratory (and furthermore were determined as Ki values rather than the IC_50_ values provided for the parasite proteases). We nonetheless felt it was valuable to include this historical cathepsin data as it provides some qualitative indication of the potential selectivity of the analogs described.

The market forces that spur new pharmaceutical development in the developed world are typically absent in the case of tropical parasitic diseases, despite indisputable medical need. One strategy to address this discrepancy is to focus on anti-parasitic drug targets that are homologous to other targets that are being actively pursued in the industry. As demonstrated here, this approach can produce potent and cell-active lead compounds directly out of a primary screening campaign. Many of these compounds are already good candidates for initial evaluation in animal pharmacokinetic and pharmacodynamic studies. Progress towards the identification of viable clinical candidates would require a robust lead optimization campaign to improve in vivo efficacy and address any safety issues that emerge. This work is far from trivial and will require significant resources in synthetic chemistry and animal pharmacology and toxicology. Providing encouragement for such work, however, is the knowledge that nitrile- and ketone-based cathepsin inhibitors not unlike those described herein have been successfully progressed into human clinical trials for other therapeutic indications.
